# Effects of G-gene Deletion and Replacement on Rabies Virus Vector Gene Expression

**DOI:** 10.1371/journal.pone.0128020

**Published:** 2015-05-29

**Authors:** Sho Sato, Shinya Ohara, Ken-Ichiro Tsutsui, Toshio Iijima

**Affiliations:** Division of Systems Neuroscience, Tohoku University Graduate School of Life Sciences, Sendai, Japan; Thomas Jefferson University, UNITED STATES

## Abstract

The glycoprotein-gene (G gene) -deleted rabies virus (RV) vector is a powerful tool to examine the function and structure of neural circuits. We previously reported that the deletion of the G gene enhances the transgene expression level of the RV vector. However, the mechanism of this enhancement remains to be clarified. We presume that there are two possible factors for this enhancement. The first factor is the glycoprotein of RV, which shows cytotoxicity; thus, may cause a dysfunction in the translation process of infected cells. The second possible factor is the enhanced expression of the L gene, which encodes viral RNA polymerase. In the RV, it is known that the gene expression level is altered depending on the position of the gene. Since G-gene deletion displaces the L gene in the genome, the expression of the L gene and viral transcription may be enhanced. In this study, we compared the transgene expression level and viral transcription of three recombinant RV vectors. The effect of glycoprotein was examined by comparing the viral gene expression of G-gene-intact RV and G-gene-replaced RV. Despite the fact that the L-gene transcription level of these two RV vectors was similar, the G-gene-replaced RV vector showed higher viral transcription and transgene expression level than the G-gene-intact RV vector. To examine the effect of the position of the L gene, we compared the viral gene expression of the G-gene-deleted RV and G-gene-replaced RV. The G-gene-deleted RV vector showed higher L-gene transcription, viral transcription, and transgene expression level than the G-gene-replaced RV vector. These results indicate that G-gene deletion enhances the transgene expression level through at least two factors, the absence of glycoprotein and enhancement of L-gene expression. These findings enable investigators to design a useful viral vector that shows a controlled desirable transgene expression level in applications.

## Introduction

Rabies virus (RV) is a nonsegmented negative-strand RNA-virus that belongs to the genus *Lyssavirus* of the family *Rhabdoviridae*. The genome of the RV encodes five viral proteins: nucleoprotein (N), phosphoprotein (P), matrix protein (M), glycoprotein (G), and RNA polymerase (L) [1]. This neurotropic virus selectively infects neurons, not glia cells, in the nervous system, and moves from neuron to neuron via synapse exclusively in a retrograde manner. This feature makes the RV and its vector a useful tool to reveal the hierarchical connectivity in complex neuronal circuits [2–6].

The infection properties of the RV are determined by its glycoprotein (RV-G), which forms spike-like projections on the surface of the viral particle that bind to neural receptors. The RV vector whose G gene was deleted from its genome (ΔG-RV) does not propagate trans-synaptically from the initial infected neurons because ΔG-RV-infected neurons do not produce infectious viral particles with RV-G [7–10]. This feature makes the ΔG-RV non-pathogenic and benefits investigators in terms of safety compared with the G-gene-intact RV. Another major advantage of G-gene deletion in the RV is that it enables selective infection of the RV vector to genetically targeted neurons by substituting the glycoprotein of the RV with that of the avian sarcoma/leukosis virus subtype A and supplying its receptor gene *in trans* within the targeted neurons. Furthermore, G-gene deletion also enables investigators to control trans-synaptic spread to presynaptic neurons by supplying the G gene within the initial infected neurons [11]. In addition to these useful features as a neural tracer, the ΔG-RV variants, which express a genetically encoded calcium indicator or optogenetic tool, have recently been developed for investigating the function of neural circuits [12].

For such purposes, a sufficient expression level of the transgene is required to detect enough signals from the ΔG-RV-infected cells or to manipulate their activity. We previously demonstrated that G-gene deletion from a G-gene-intact RV vector enhances the expression level of the transgene, which encodes a monomeric red fluorescent protein (mRFP) [13]. However, the reason of the enhancement remains to be clarified. We presume that there are at least two possible factors for the enhancement of the transgene expression level of the ΔG-RV. The first possible factor is the absence of the RV-G. We previously reported that the RV-G affects the cell viability and resting membrane potential of infected cells [13]. It is possible that the cytotoxicity of the RV-G causes some dysfunction in the translation process of infected cells. The other possible factor is the increased expression of the L gene, which encodes the viral polymerase. It was previously reported that increased L-gene expression enhances viral mRNA transcription [14]. In nonsegmented negative-strand RNA viruses, including the RV, it has been shown that the expression levels of the viral genes decrease monotonically as the distance increases from the start (3’ end) of the genome [15–17]. Since G-gene deletion displaces the L gene anteriorly in their genome, the expression level of the L gene in ΔG-RV-infected cells is presumed to be higher than that of the L gene in G-gene-intact RV-infected cells. This increase in the L-gene expression level might enhance the viral transgene expression in ΔG-RV-infected cells. In this study, we compared the transgene expression level and viral transcription of three RV vectors, G-gene-intact RV, G-gene-deleted RV and G-gene-replaced RV. The G-gene-replaced RV was created by replacing the RV-G with a fluorescent protein-encoding gene which was assumed that the fluorescent protein-encoding gene itself would not have any noteworthy effects on the viral gene expression. The aim of the replacement was to create G-gene-deleted RV without changing the position of the L gene in the genome. To examine the effect of the RV-G, we compared the viral gene expression of the G-gene-intact and G-gene-replaced RVs. The effect of the position of the L gene was investigated by comparing the viral gene expression of the G-gene-deleted and G-gene-replaced RVs.

## Materials and Methods

### Plasmid construction

The full-length genome plasmids of the G-gene-deleted RV (rHEP5.0-ΔG-mRFP, DDBJ/GenBank/EMBL accession number, AB839170) was constructed by deleting the entire G gene from the G-gene-intact RV (pHEP5.0-CVSG-mRFP, accession number, AB839169), as described previously [13]. The G-gene-replaced RV (pHEP5.0-ΔG-mRFP-BPB) was constructed by replacing the RV-G of pHEP5.0-CVSG-mRFP with two blue fluorescent proteins (TagBFP, Evrogen) [18] linked by a P2A self-cleavage sequence [19] (BPB) to rHEP5.0-CVSG-mRFP. Two PCR fragments containing the TagBFP sequence were inserted in the synthesized sequence 5’-CCTTAAGG(1)CCGCGG(2)**GGAAGCGGAGCTACTAACTTCAGCCTGCTGAAGCAGGCTGGAGACGTGGAGGAGAACCCTGGACCT** ACCGGT(3)TCTAGA(4)-3’ (the Afl II (1), Sac II (2), Age I (3), and Xba I (4) sites are underlined, the P2A sequence is shown in bold) by using the following primers: for the first TagBFP fragment, 5’-ACCCTTAAGGAAAGATGAGCGAGCTGATTAAGGAGA-3’ (the Afl II site is underlined) and 3’-GGTCCGCGGATTAAGCTTGTGCCCCAGTTTGCT-5’ (the Sac II site is underlined), and for the second TagBFP fragment, 5’-ATTACCGGTACCATGAGCGAGCTGATTAAGGAGAA-3’ (Age I site is under lined) and 3’-GGTTCTAGA(1)CGTACG(2)AGAGGTGTTAGTTTTTTTCCCCGGG(3)TTTAATTAAGCTTGTGCCCCAGTTTGCT-5’ (the Xba I (1), BsiW I (2), and Xma I (3) sites are underlined). The BPB sequence was inserted into pHEP5.0-CVSG-mRFP by Afl II and BsiW I.

### Virus recovery

The pHEP5.0-ΔG-mRFP, pHEP5.0-CVSG-mRFP, and pHEP5.0-ΔG-mRFP-BPB were rescued using mouse neuroblastoma cells of A/J mouse origin (NA), as previously described [20]. The rescued viruses generated from pHEP5.0-ΔG-mRFP, pHEP5.0-CVSG-mRFP, and pHEP5.0-ΔG-mRFP-BPB were designated as rHEP5.0-ΔG-mRFP, rHEP5.0-CVSG-mRFP, and rHEP5.0-ΔG-mRFP-BPB, respectively. Since rHEP5.0-ΔG-mRFP and rHEP5.0-ΔG-mRFP-BPB do not express G, the NA cell line expressing the G of the Challenge Virus Standard (CVS) strain was used for the inoculation of this virus. To establish this cell line, NA cells were transfected with pHN-HP-CVSG plasmid. This plasmid contains the N and P protein of HEP strain and G protein of CVS strain coding DNA sequences linked by 2A sequence under the CMV promoter. This plasmid also encodes the Zeocin resistance gene. The transfected cells were selected according to Zeocin (Life Technologies) resistance during the course of four weeks. The resistance cells were cloned and examined for infectious virion production ability. The viruses were concentrated using centrifugal filter devices (Amicon Ultra-15), and viral suspensions were kept in small aliquots at −80°C. Each aliquot was thawed in a safety cabinet before each experiment. To determine the viral titer, we conducted a direct fluorescent test using NA cells, as previously described [21].

### Comparison of mRFP expression level in infected cells

The NA cells were grown on glass coverslips in a 24-well plate and maintained at 34°C in minimum essential medium supplemented with 10% heat-inactivated fetal bovine serum (FBS). To measure the mRFP expression level of the three RV vectors, each of the three viruses was applied to the NA cells at a multiplicity of infection (m.o.i) of 20. To confirm that MOI 20 was enough to achieve saturated infection, we performed immunostaining with anti-N antibody at 12 hours after infection and confirmed that over 97% of cells were infected by the initial infection of RV after using the infection of an MOI 10. One day after infection, the medium was replaced with a fresh one and the cells were incubated at 34°C. Three and 6 days after infection, the cells were fixed for 45 min at 4°C in phosphate-buffered saline (PBS) containing 4% formaldehyde. The fluorescence of the infected cells was detected using the confocal laser-scanning microscope LSM 5 Exciter (Carl Zeiss). The average fluorescent intensity of mRFP was evaluated by measuring the average fluorescent intensity per pixel of the infected cells with ImageJ software (http://rsb.info.nih.gov/ij). We measured the fluorescence intensity only from cells in which fluorescence was above the fixed threshold; cells that showed any fluorescence were not included in the analysis.

### Comparison of viral transcription in infected cells

To minimalize the effect of cell proliferation to the transcription, all NA cells for the RNA quantification were used after the cells reached 100% confluence and were incubated at 34°C. The NA cells were grown on 48-well plates, and the three viruses were applied at an m.o.i of 10. One day after infection, the medium was replaced with a fresh one and cells were incubated at 34°C. Total RNA from RV-infected NA cells was collected 1, 3 and 6 days post infection (dpi) with the RNeasy minikit (Qiagen).

#### Northern blot analysis of viral transcripts

Northern blot analysis was conducted using the protocol “DIG Application Manual for Filter Hybridization” (Roche). For northern blot hybridization analysis, the RNAs were electrophoresed on 1.2% agarose gel in 20 mM 3-Morpholinopropanesulfonic acid, 5 mM Sodium acetate 2 mM EDTA containing 2% formaldehyde. After electrophoresis, the RNAs were transferred onto a positively charged nylon membrane (Roche), then the membrane was hybridized with a DIG-labeled gene-specific probe (N, mRFP, P, M, G, BPB, or L gene). The DIG labeled gene-specific probes were amplified using the following primers: for the N gene, 5’-CGTACTGATGTAGAAGGGAATTGG-3’ and 3’-CCCTGGCTCAAACATTCTCCTTATC-5’, for the mRFP gene, 5’-CAAGCTGAAGGTGACCAAGG-3’ and 3’-GTTGTGGGAGGTGATGTCCAG-5’, for the P gene, 5’-GAACCCATAGAAGTGGACAATCTC-3’ and 3’-ATGAGCGATCTCAGCCTCTAC-5’, for the M gene, 5’-AAACTGTAGGGATGAGGACACC-3’ and 3’-GATACACCAGATCCTGCCTTG-5’, for the CVSG gene, 5’-AGGTGTTGTGACAGAGGCAGAGAC-3’ and 3’-TTTCATCCACAAAGCCGCAAG-5’, for the BPB gene 5’-CCCGCTAAGAACCTCAAGATG-3’ and 3’-AGGTCTTGCTGCCGTAGAG-5’, and for the L gene, 5’-GATTCCTTGCCCGAGACCAC-3’ and 3’-GTCTGTCATAGTCAGTGCGTC-5’. The probe-target hybrids on the membrane were visualized with disodium 3-(4-methoxyspiro {1,2-dioxetane-3,2'-(5'-chloro) tricyclo [3.3.1.13,7]decan}-4-yl)phenyl phosphate (CSPD). The luminescent images were detected using Image Quant 400 (GE Healthcare) with different exposure times for each lane. RNA constructions were estimated from the size of transcripts, and the relative quantities of the transcripts were calculated using the imageJ gel analysis tool for each lane.

#### Quantitative real-time PCR analysis of viral transcripts

For quantitative real-time PCR, the collected total RNAs were treated with DNaseI (NEB). The RNAs were reverse transcribed into cDNA by using the PrimeScript 1st strand cDNA Synthesis Kit (Takara) using oligo dt primer according to the manufacturer’s protocol. Quantitative real-time PCR analysis was conducted on the Light Cycler nano (Roche) using the SYBR Green Realtime PCR Master Mix-Plus (TOYOBO LIFE SCIENCE) according to the manufacturer’s protocol. The following primers were used to determine the amount of mRNA of each gene: for the RPL13A gene, 5’-ATGACAAGAAAAAGCGGATG-3’ and 3’-CTTTTCTGCCTGTTTCCGTA-5’, for the N gene, 5’-AGTCGGTCTTCTCCTGAGTCTGTAC-3’ and 3’-GTTGTCATCAGGGTGTGGTGTTC-5’, for the mRFP gene, 5’-CCTGAAGGGCGAGATCAAGATGAG-3’ and 3’-TAGTCCTCGTTGTGGGAGGTGATG-5’, for the P gene, 5’-CCCAACCTCCTGTTCCAATCGTAC-3’ and 3’-TCCTGGAGGGTTAGGAAAGTTGAC-5’, for the M gene, 5’-ATGTCCCGCTGAAAGAACTCACAAG-3’ and 3’-TGAGTAACCGTTCGGACTGCAC-5’, for the G gene, 5’-AGGTGTTGTGACAGAGGCAGAGAC-3’ and 3’-ATCTTCCAGTTATACGCGGCTCTAC-5’, for the BPB gene 5’-GGGAAGCGGAGCTACTAAC-3’ and 3’-TAGGTCCAGGGTTCTCCTCC-5’, and for the L gene 5’-AACAGGAGCCGGAGATGTATAACC-3’ and 3’-ATCGACCCTCTCAAGGCAACTTAAC-5’.

All samples were run in triplicate. The specificity of products of the SYBR Green reactions were validated by dissociation curves after quantification. The relative amounts of viral-gene mRNA were calculated based on the calibration curve which was produced from serial 10-fold dilutions of cDNA of known copy number and normalized based on the amount of the RPL13A gene mRNA of the same sample. The amounts were collected for “the mean value of the amounts of L-gene-coding mRNA of rHEP5.0-CVSG-mRFP-infected cells 1 dpi” to equal 1.

## Results

**Fig 1:**
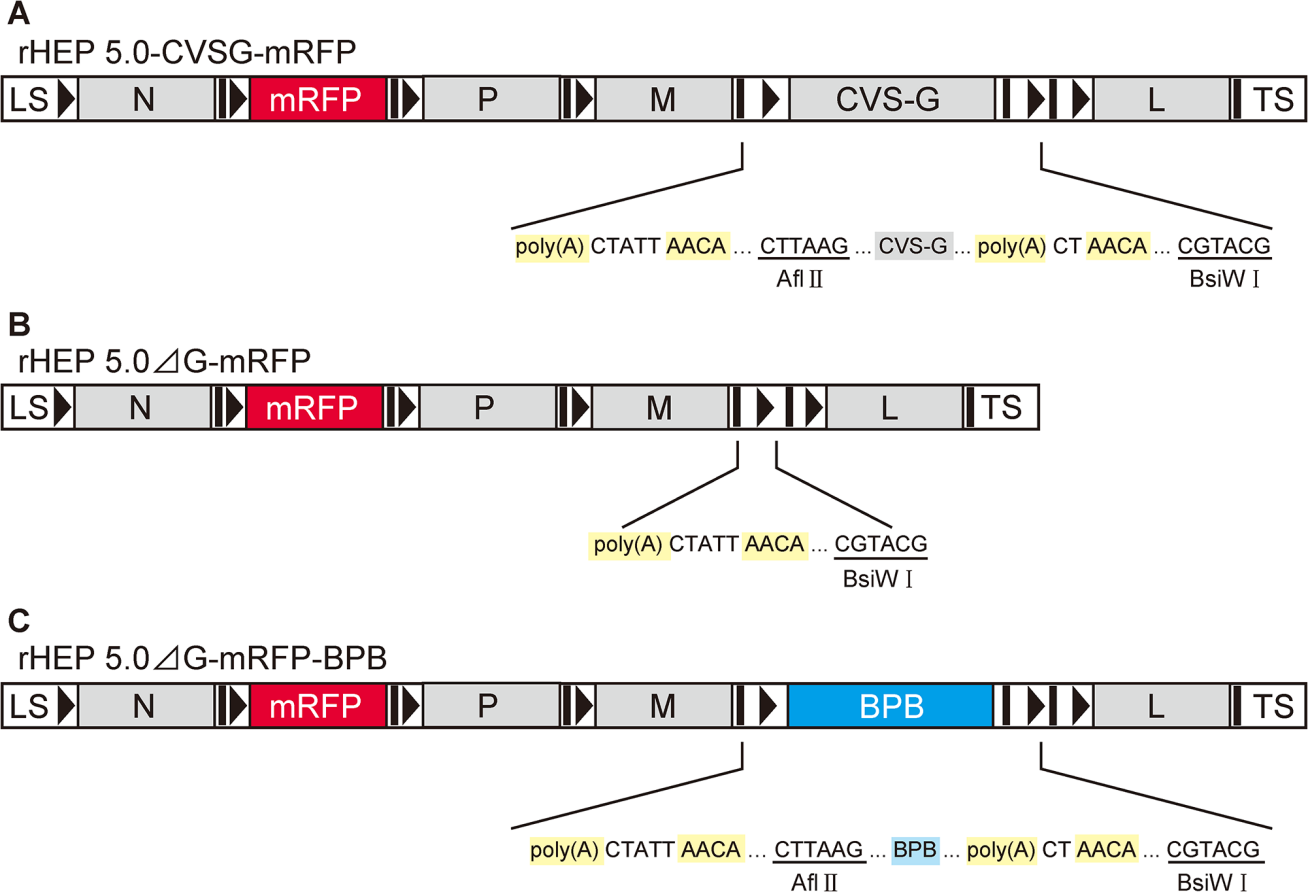
Genome organization of three RV vector variants. Genome organization and the close-up between the end of the M gene and BsiW I site. The transcription unit, start and stop/polyadenylation signals, AACA, and poly(A) are respectively shown as black bars and black arrowheads in the schematic diagram and are shaded yellow in the close-up. LS: leader sequence; TS: trailer sequence. **A**: rHEP5.0-CVSG-mRFP was constructed based on HEP strain by the replacement of the G gene with the G gene derived from the CVS strain (CVS-G). rHEP5.0-CVSG-mRFP has an mRFP gene as a transgene between the N and P genes and has an additional transcription unit between the G and L genes. **B**: In rHEP5.0-ΔG-mRFP, the G gene and a transcription unit were deleted from rHEP5.0-CVSG-mRFP. **C**: rHEP5.0-ΔG-mRFP-BPB had two TagBFP genes, which were linked by a P2A sequence (BPB) as one gene in the place of the G gene.

We examined the effects of G-gene deletion and that of G-gene replacement with a fluorescent protein-encoding gene on the transgene expression level and the viral mRNA transcriptions. Based on the attenuated high egg passage-flury (HEP) strain, we created three variants of RV vectors, which express mRFP as a transgene (Fig 1). The first RV vector, G-gene-intact RV (rHEP5.0-CVSG-mRFP), was created by exchanging the G gene of the HEP strain with that of the CVS strain, which has been generally used for tracing experiments. We previously reported that this G-gene-exchanged RV vector shows neuron-specific retrograde infection similar to the CVS strain [22]. The second RV vector, G-gene-deleted RV (rHEP5.0-ΔG-mRFP), was created by deleting the G gene from rHEP5.0-CVSG-mRFP. The third RV vector, G-gene-replaced RV (rHEP5.0-ΔG-mRFP-BPB), was created by replacing the G gene with two TagBFP connected by a P2A sequence as a single gene. Since the length of the two TagBFP and the P2A sequence (1482 nt) is nearly equal to that of the CVS-G (1575 nt), the L genes of rHEP5.0-CVSG-mRFP and rHEP5.0-ΔG-mRFP-BPB are located at a similar position in their genomes. To examine the effect of the RV-G, we compared the transgene expression level and viral mRNA transcriptions of the rHEP5.0-CVSG-mRFP- and rHEP5.0-ΔG-mRFP-BPB-infected cells. The effect of the position of the L gene in the genome was investigated by comparing the transgene expression level and viral mRNA transcriptions of the rHEP5.0-ΔG-mRFP and rHEP5.0-ΔG-mRFP-BPB in the infected cells.

### Comparison of mRFP-expression level in infected cells

**Fig 2:**
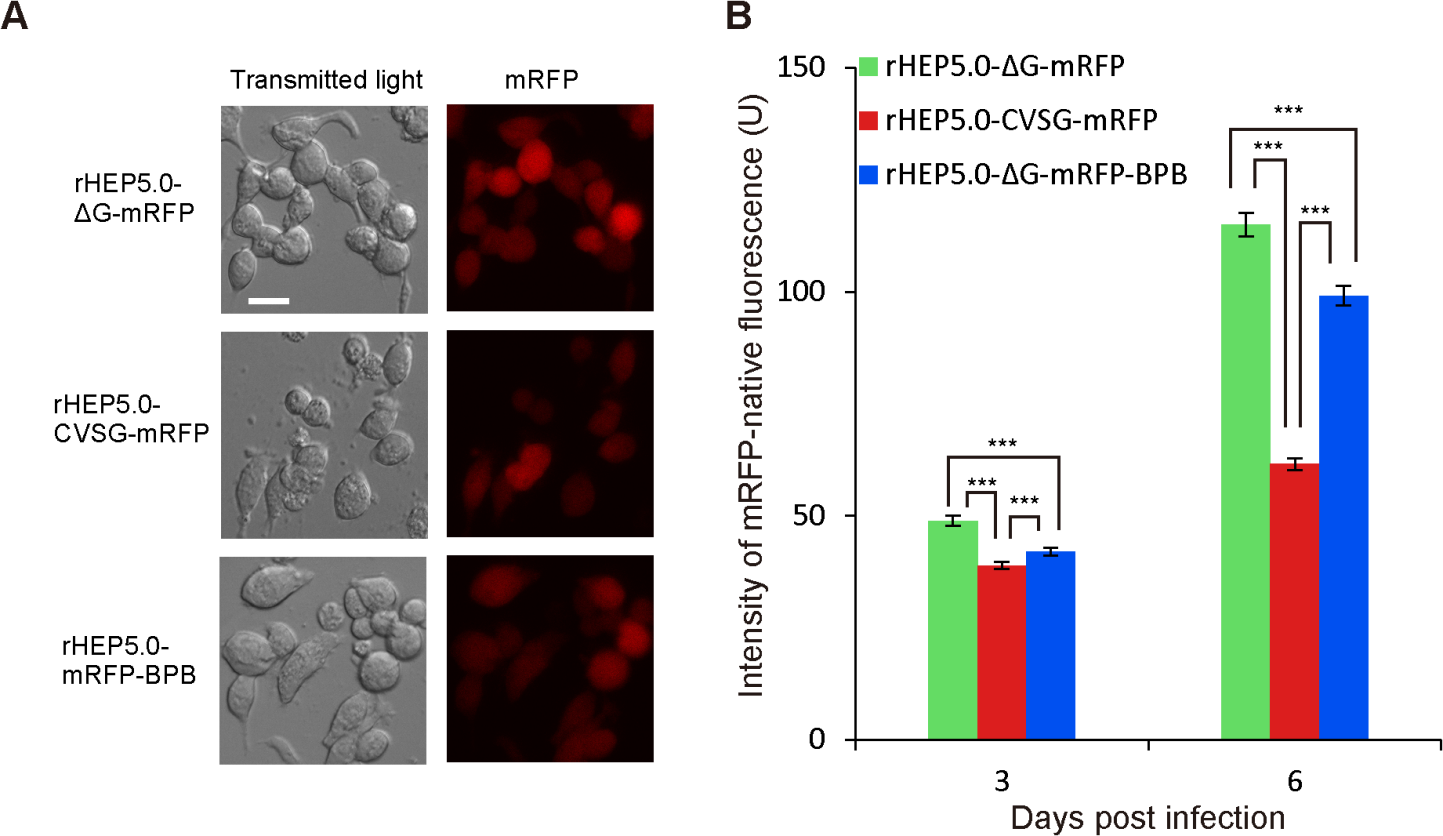
Transgene expression of three RV vectors in infected NA cells. **A**: Photomicrographs of the RV-vector-infected NA cells 6 dpi. The three RV vector expressed the transgene mRFP, which was inserted to the N and P genes. Scale bar = 20 μm. **B**: The mean fluorescence intensity of mRFP in the RV-vector-infected NA cells (mean ± SEM, *** p < 0.001). The G-gene-deleted RV-vector rHEP5.0-ΔG-mRFP-infected cells showed a significantly higher mRFP expression level in relation to the G-gene-intact RV rHEP5.0-CVSG-mRFP-infected cells and G-gene-replaced RV-vector rHEP5.0-ΔG-mRFP-BPB-infected cells. The expression level of mRFP in the rHEP5.0-ΔG-mRFP-BPB-infected cells was significantly higher than that of the rHEP5.0-CVSG-mRFP-infected cells.

The mRFP-expression levels of rHEP5.0-ΔG-mRFP, rHEP5.0-CVSG-mRFP, and rHEP5.0-ΔG-mRFP-BPB were compared in the NA cells. The NA cells were infected with one of the three RV variants that express mRFP as a transgene (Fig 2A). The fluorescent intensity of mRFP was examined at different points in time (3 and 6 dpi) (Fig 2B). At 3 dpi, the fluorescent intensity of mRFP of the rHEP5.0-ΔG-mRFP-infected cells was higher than that of the rHEP5.0-CVSG-mRFP- and rHEP5.0-ΔG-mRFP-BPB-infected cells (Kruskal-Wallis rank sum test; Mann-Whitney U test, Holm-corrected, p < 0.001), and the fluorescent intensity of mRFP of the rHEP5.0-ΔG-mRFP-BPB-infected cells was high in comparison with that of the rHEP5.0-CVSG-mRFP-infected cells (p < 0.001). At 6 dpi, the differences in the fluorescent intensity expanded over time after infection and the quantitative relation of the fluorescence intensity remained unchanged (p < 0.001, all pairs at 6 dpi). These results showed that the absence of G, after the G-gene deletion and its replacement, affected the expression level of the mRFP gene, which was inserted between the N and P genes in the RV genome, and the effect of the deletion was stronger than that of the replacement.

### Comparison of viral transcription in infected cells

To determine the effect of G-gene deletion and its replacement on the viral transcription, we conducted northern blot and quantitative real-time PCR analysis of RV viral mRNA. It was previously reported that the RV HEP strain produces multicistronic transcripts; mRNAs containing more than one gene sequence [23]. Since the multicistronic transcripts hybridize to more than one pair of viral-gene-specific primers, the amounts of multicistronic transcripts are counted more than once in quantitative real-time PCR. For instance, a tricistronic mRNA, which contains the sequences of the mRFP, P and M genes, would be counted three times as an mRFP-, P-, and M-gene mRNA in quantitative real-time PCR. Within a multicistronic transcript, it is thought that only the first cistron of the multicistronic transcript is normally translated, but not the downstream cistron [24]; thus, this mRNA is translatable into mRFP but not into the P or M protein. For these reasons, we estimated the valid amounts of viral mRNAs assumed translated into the viral proteins (the amounts of “gene-coding mRNAs”). First, we evaluated the total amounts of viral gene mRNAs that have the target gene sequence (“gene-related RNAs”, Table 1) by quantitative real-time PCR using each viral-gene-specific primer pair and normalized the amounts by the amount of housekeeping RPL13A-gene mRNA. Second, we evaluated the percentage of the gene-coding mRNA in the gene-related RNA by using the northern blot analysis. Finally, the amount of gene coding RNA was calculated as the product of the amount of gene-related RNA and percentage of gene-coding mRNA.

**Table 1:** Relative amounts of gene-related RNA.

Gene	Date	rHEP5.0-ΔG-mRFP	rHEP5.0-CVSG-mRFP	rHEP5.0-ΔG-mRFP-BPB
N	1dpi	21.9 ± 2.9	8.1 ± 0.7	14.1 ± 0.9
	3dpi	97.5 ± 9.7	43.8 ± 5.9	74.5 ± 10.0
	6dpi	139.1 ± 20.3	42.5 ± 5.9	77.9 ± 5.4
mRFP	1dpi	10.9 ± 2.1	5.4 ± 0.6	7.1 ± 0.8
	3dpi	75.0 ± 7.8	38.7 ± 5.6	55.5 ± 7.4
	6dpi	146.2 ± 23.0	54.8 ± 11.3	67.8 ± 3.9
P	1dpi	12.0 ± 1.7	4.7 ± 0.4	7.2 ± 0.5
	3dpi	45.6 ± 3.2	27.2 ± 3.3	34.9 ± 4.0
	6dpi	87.3 ± 8.2	31.4 ± 5.6	44.8 ± 3.4
M	1dpi	5.2 ± 0.7	2.2 ± 0.1	2.9 ± 0.2
	3dpi	17.7 ± 1.5	10.8 ± 1.6	12.9 ± 1.7
	6dpi	49.1 ± 6.6	17.9 ± 4.1	22.6 ± 0.9
G(BPB)	1dpi	-	2.6 ± 0.3	2.9 ± 0.4
	3dpi	-	8.6 ± 1.5	8.8 ± 1.0
	6dpi	-	7.5 ± 2.2	22.6 ± 0.4
L	1dpi	3.4 ± 0.4	1.0 ± 0.1	1.4 ± 0.1
	3dpi	17.3 ± 3.5	8.8 ± 1.7	7.9 ± 1.2
	6dpi	9.8 ± 2.2	11.4 ± 3.5	4.7 ± 0.3

The relative amounts of gene-related RNA were estimated from the results of quantitative PCR using each gene specific primer pairs (mean ± SEM). The amounts were collected for “the mean value of the amounts of L-gene-related mRNA of rHEP5.0-CVSG-mRFP-infected cells 1 dpi” to equal 1.

#### Northern blot analysis of viral transcripts

The total RNA of the RV-infected NA cells was collected at different points in time (1, 3 and 6 dpi), and viral RNA transcripts were examined through northern blot analysis (S1 Fig). As we expected, the three RV vectors, which were created based on the HEP strain, produced multicistronic transcripts at a considerable rate (S1 Table). All viral RNA transcripts were quantified by measuring the intensity of all bands, as shown in S1 Fig, and the percentages of gene-coding mRNA in gene-related mRNA were calculated (Table 2). It was estimated that over 85% of the L-gene-related mRNA was the L-gene-coding mRNA in the rHEP5.0-ΔG-mRFP-infected cells 1, 3 and 6 dpi, and this percentage was higher than those of L-gene-coding mRNA in the rHEP5.0-CVSG-mRFP- (58.7–81.2%) and rHEP5.0-ΔG-mRFP-BPB-infected cells (73.0–87.4%) at the three time points. We observed that the percentages of gene-coding mRNA changed with time after infection in some genes, for example, the percentage of the P-gene-coding mRNA decreased as time passed in the three RV-infected cells.

**Table 2:** Percentages of gene-coding mRNA in gene-related RNA.

Gene	Date	rHEP5.0-ΔG-mRFP	rHEP5.0-CVSG-mRFP	rHEP5.0-ΔG-mRFP-BPB
N	1dpi	100.0 (%)	100.0	100.0
	3dpi	100.0	100.0	100.0
	6dpi	100.0	100.0	100.0
mRFP	1dpi	73.8	82.7	88.4
	3dpi	83.7	87.0	89.6
	6dpi	79.1	80.5	88.6
P	1dpi	79.9	78.8	79.5
	3dpi	70.6	71.1	62.0
	6dpi	65.4	56.2	59.5
M	1dpi	50.5	78.9	60.6
	3dpi	50.9	58.4	58.3
	6dpi	50.5	48.3	68.0
G(BPB)	1dpi	-	57.0	70.9
	3dpi	-	68.8	67.0
	6dpi	-	65.1	72.7
L	1dpi	91.3	81.2	87.4
	3dpi	85.9	69.0	74.2
	6dpi	87.8	58.7	73.0

The percentages of the translatable gene-coding mRNA in the gene-related RNA were calculated based on the results of northern blot analysis (S1 Fig). Multicistronic mRNAs were sorted by the first cistron, for example the dicistronic RNA, which contains the sequences of N and P genes (N+P), was sorted as N-gene-coding mRNA but not as P-gene-coding mRNA.

#### Quantitative real-time PCR analysis of viral transcripts

**Fig 3:**
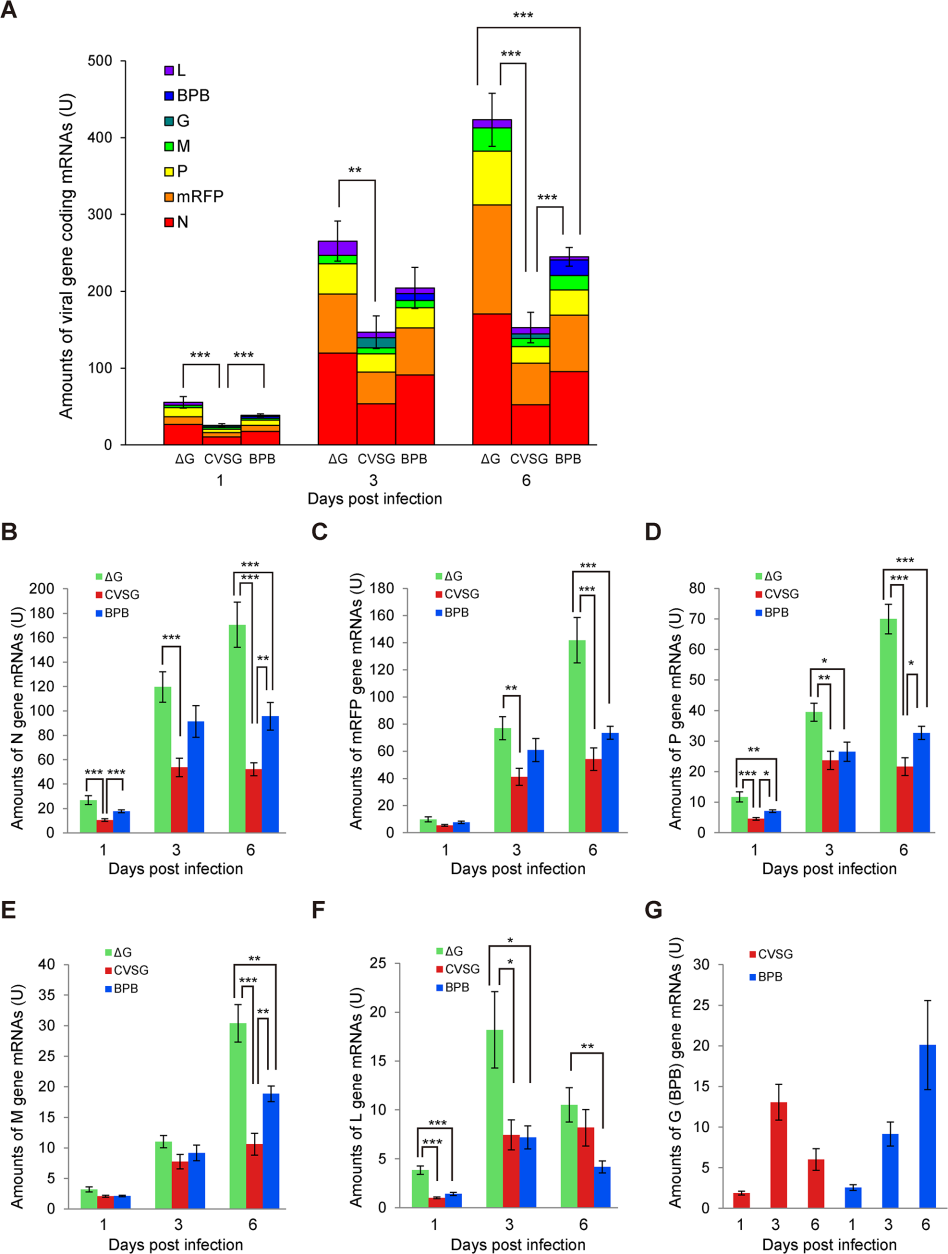
Quantitative analysis of viral mRNA. The relative amounts of viral gene-coding mRNAs were assayed by real-time PCR and Northern blot analysis (mean ± SEM, n = 10 for each condition, * p < 0.05, ** p < 0.01, *** p < 0.001). ΔG, rHEP5.0-ΔG-mRFP; CVSG, rHEP5.0-CVSG-mRFP; BPB, rHEP5.0-ΔG-mRFP-BPB. **A**: The sum of the viral gene-coding mRNAs in the RV-vector-infected NA cells at different time points. The quantitative relation of the viral gene-coding mRNAs in the three RV infected cells was identical to that of the mRFP fluorescence intensity (Fig 2) 6 dpi. **B-G**: The amounts of viral gene-coding mRNAs of **(B)** N, **(C)** mRFP, **(D)** P, **(E)** M, **(F)** L, and **(G)** CVS-G and BPB genes at different time points. The quantitative relations of the N, P, and M gene-coding mRNAs in the three RV infected cells were the same as that of the sum of the viral gene-coding mRNAs and that of the mRFP fluorescence intensity 6 dpi. The amount of L-gene-coding mRNA in rHEP5.0-ΔG-mRFP-infected cells was significantly larger than that of the other two RV-infected cells 1 and 3 dpi.

Next, we quantified and compared the amount of each viral-gene-coding mRNA in the cells infected with one of the three RV variants (Fig 3A, S2 Table). The quantitative relation of the sum of viral-gene-coding mRNAs was similar to the result of the fluorescent intensity comparison described above 6 dpi. The sum of the gene-coding mRNAs of the rHEP5.0-ΔG-mRFP-infected cells was higher than those of the rHEP5.0-CVSG-mRFP- and rHEP5.0-ΔG-mRFP-BPB-infected cells, and the sum of the gene-coding mRNAs of the rHEP5.0-ΔG-mRFP-BPB-infected cells was larger than that of the rHEP5.0-CVSG-mRFP-infected cells (Kruskal-Wallis rank sum test; Mann-Whitney U test, Holm-corrected. p < 0.001, all pairs 6 dpi). The amounts of N-, P- and M-gene-coding mRNAs showed this quantitative relation in common, and the amount of mRFP-gene-coding mRNAs showed a similar tendency (Fig 3B, 3C, 3D and 3E).

The three RV vectors showed notable differences in the amounts of L-gene-coding mRNAs (Fig 3F). At 1 and 3 dpi, as expected, the amount of L-protein-coding mRNA in the rHEP5.0-ΔG-mRFP-infected cells was significantly larger than those of the rHEP5.0-CVSG-mRFP- and rHEP5.0-ΔG-mRFP-BPB-infected cells (p < 0.001 at 1 dpi, p < 0.05 3 dpi). Most of the other viral-gene-coding mRNAs monotonically increased over time after infection, whereas the amounts of the L-gene-coding mRNAs in the rHEP5.0-ΔG-mRFP- and rHEP5.0-ΔG-mRFP-BPB-infected cells decreased 6 dpi. The significant difference between the amounts of the L-gene-coding mRNAs in the rHEP5.0-ΔG-mRFP- and rHEP5.0-CVSG-mRFP-infected cells was not observed (rHEP5.0-ΔG-mRFP vs. rHEP5.0-CVSG-mRFP p = 0.436, rHEP5.0-ΔG-mRFP vs. rHEP5.0-ΔG-mRFP-BPB p = 0.0086). No significant difference in the amounts of L-protein-coding mRNAs was observed between the rHEP5.0-CVSG-mRFP- and rHEP5.0-ΔG-mRFP-BPB-infected cells 1, 3 and 6 dpi.

**Fig 4:**
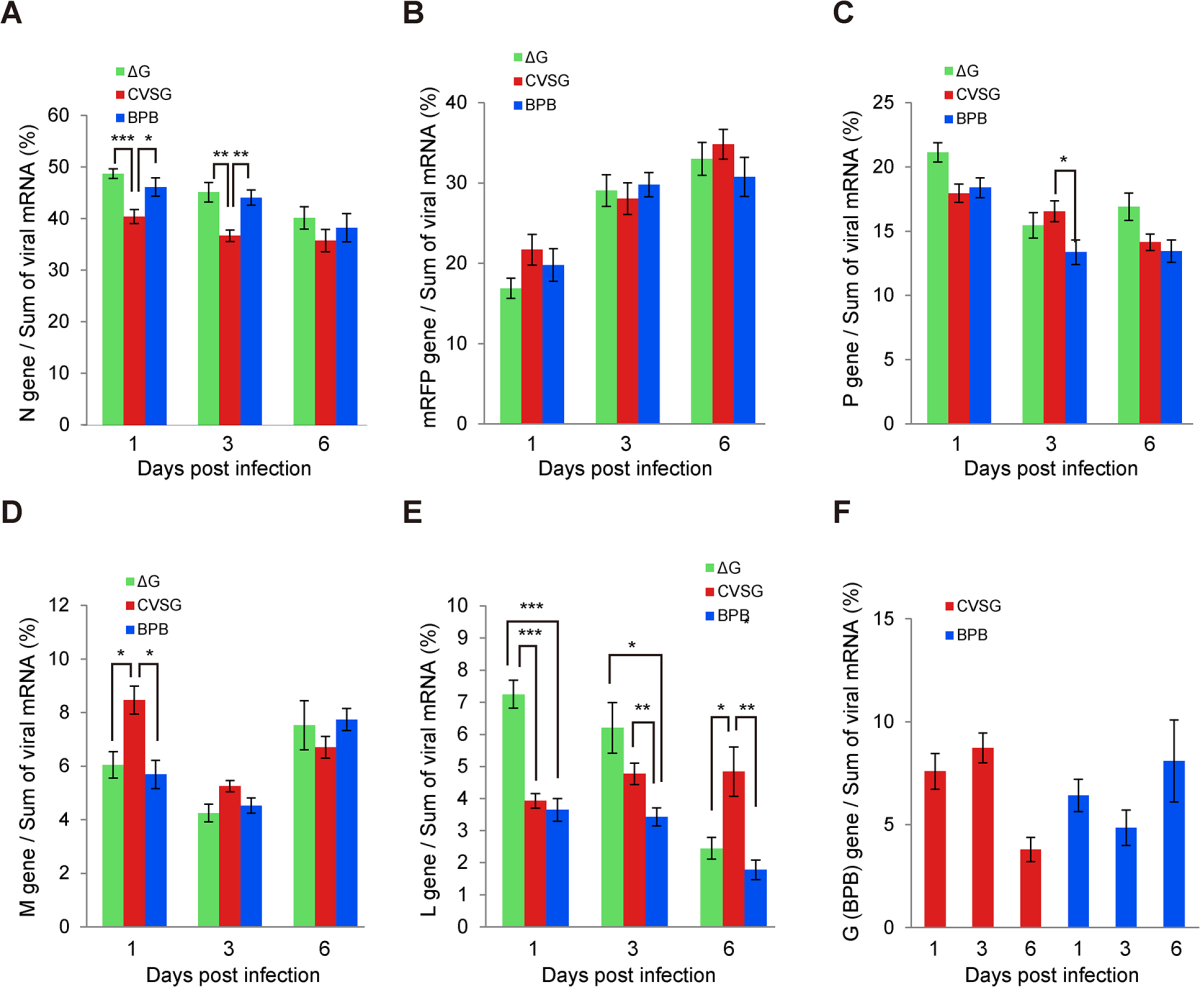
Percentages of viral gene-coding mRNA. The percentages of viral gene-coding mRNAs from the sum of the viral gene-coding mRNAs (mean ± SEM, n = 10 for each condition, * p < 0.05, ** p < 0.01, *** p < 0.001). **A**: N gene, **B**: mRFP gene, **C**: P gene, **D**: M gene, **E**: L gene, and **F**: CVS-G and BPB genes. The percentages of L-gene-coding mRNA in the rHEP5.0-ΔG-mRFP and rHEP5.0-ΔG-mRFP-BPB-infected cells decreased as time passed after infection.

We examined the percentage of each viral-gene-coding mRNA from the sum of viral-gene-coding mRNAs (Fig 4). As is the case in quantitative analysis, the three RV vectors showed discriminating results in the percentages of L-gene-coding mRNAs (Fig 4E). At 1 dpi, the percentage of L-gene-coding mRNA from the sum of viral-gene-coding mRNAs, which were produced in the rHEP5.0-ΔG-mRFP-infected cells, was higher than that of the other two RVs (p<0.001). The percentages of L-gene-coding mRNAs in the rHEP5.0-ΔG-mRFP- and rHEP5.0-ΔG-mRFP-BPB-infected cells decreased from 1 to 6 dpi, but that of the rHEP5.0-CVSG-mRFP-infected cells did not. The percentage of L-protein-coding mRNA from the sum of the viral mRNAs in the rHEP5.0-CVSG-mRFP-infected cells was higher than those of the other two RV-infected cells 6 dpi (rHEP5.0-ΔG-mRFP vs. rHEP5.0-CVSG-mRFP p < 0.05, rHEP5.0-CVSG-mRFP vs. rHEP5.0-ΔG-mRFP-BPB p < 0.01). Although some significant differences were observed in the percentages of the gene-coding mRNAs from the sum of viral-gene-coding mRNAs regarding the N, P, and M genes, the difference was not as notable as that of the L gene (Fig 4A, 4C and 4D).

These results showed that G-gene deletion significantly increased the L-gene-coding mRNA 1 and 3 dpi and activated the viral transcription. On the other hand, the replacement of the G gene did not increase the L-gene-coding mRNA; nevertheless, the replacement of the G gene activated the viral transcription. The effect of the replacement of the G gene on the viral transcription was smaller than that of G-gene deletion.

## Discussion

We previously reported that the G-gene deletion of the RV vector enhances the expression of the transgene, which is inserted between the N and P genes [13]. This result suggests that the RV-G affects viral transgene expression, although the RV-G has little known involvement in viral transcription and replication. We presumed that this enhancement of transgene expression may be due to two factors. The first possible factor is the reduced cytotoxicity caused by the absence of the RV-G. The second possible factor is the enhanced expression of the viral polymerase L gene caused by the displacement of the L gene in the genome after G-gene deletion.

To investigate the reasons of the transgene expression level enhancement of the RV after G-gene deletion, we examined the effects of G-gene deletion and replacement on the RV vector gene expression and viral transcription. The comparison between the characteristics of the G-gene-intact RV (rHEP5.0-CVSG-mRFP) and G-gene-replaced RV (rHEP5.0-ΔG-mRFP-BPB) in infected cells shows the effect of the presence of the RV-G, while the comparison between the characteristics of the G-gene-deleted RV (rHEP5.0-ΔG-mRFP) and rHEP5.0-ΔG-mRFP-BPB in infected cells shows the effect of the position of the L gene on the RV genome. We observed that not only G-gene deletion but also its replacement enhanced the expression of the transgene, which was inserted between the N and P genes, and the effect of G-gene deletion was larger than that of G-gene replacement. We also observed that G-gene deletion and its replacement enhanced viral transcription and G-gene deletion affected the transcription more than G-gene replacement. Additionally, we confirmed that G-gene deletion increased the L-gene-coding mRNA transcript but its replacement did not. These results suggest that the enhancement of the transgene expression level after G-gene deletion was due to at least two factors.

One factor is the expression level of the L gene, which is a 5’ terminal gene of the RV. The L gene encodes the viral RNA polymerase, and it has been reported that the overexpressed L gene increases the viral-gene mRNA transcripts and the N and P protein expression levels [14]. In this study, we observed the increase in the L-gene-coding mRNA caused by G-gene deletion at 1 and 3dpi. This can be explained by the transcription regulation mechanism of the nonsegmented negative-strand RNA viruses including the RV. In these viruses, it is believed that the expression levels of the viral genes decrease monotonically as the distance increases from the start (3’ end) of the genome [15–17]. In rHEP5.0-CVSG-mRFP and rHEP5.0-ΔG-mRFP-BPB, the L gene is the sixth gene from the start of the genome, whereas in rHEP5.0-ΔG-mRFP, the L gene is fifth from the start of the genome. Most likely, the enhanced viral transcription, which was observed after G-gene deletion, resulted from the increased viral polymerase, which was caused by the smaller number of genes in front of the L gene on the RV genome. Moreover, the northern blot analysis showed that the percentage of translatable L-gene-coding mRNA in the L gene-related mRNA of the rHEP5.0-ΔG-mRFP-infected cells was higher than those of the other two RV-infected cells. This might also contribute to the enhancement of the L-gene expression level, which was observed in the G-gene-deleted RV-vector-infected cells. We must, however, note that the L-gene-coding mRNA decreased in the rHEP5.0-ΔG-mRFP- and rHEP5.0-ΔG-mRFP-BPB-infected cells at 6 dpi, and thus, there was a discrepancy between the level of L-gene-coding mRNA and other viral-gene-coding mRNAs at this time point.

Another factor is the effects of the RV-G. By comparing the characteristics of rHEP5.0-CVSG-mRFP and rHEP5.0-ΔG-mRFP-BPB, we observed that G-gene replacement with a similar sized fluorescent protein encoding gene increased the transgene expression and viral mRNA transcripts without increasing the L-gene transcript. It is known that the RV-G largely affects cell viability [25–28], and we previously demonstrated that G-gene deletion improves the RV cytotoxicity in infected cells [13]. The lack of these effects of the RV-G might contribute to the enhanced viral gene expression in rHEP5.0-ΔG-mRFP- and rHEP5.0-ΔG-mRFP-BPB-infected cells. Additionally, we have observed that the RV variants, which do not have the G gene, and the RV variant, which has the G gene, transcribed the L gene differently. The amount of the L-gene-coding mRNA in the rHEP5.0-ΔG-mRFP- and rHEP5.0-ΔG-mRFP-BPB-infected cells was larger 3 dpi rather than 6 dpi. However, such a decrease in the L-gene-coding mRNA was not observed in the rHEP5.0-CVSG-mRFP-infected cells. This difference might be due to the effects of the RV-G on viral transcription. Little is known about the mechanism on how the RV-G affects gene expression and much remains to be clarified, but it has been reported that the G gene affects viral transcription activity [29, 30]. A possible mechanism on how the RV-G affects viral transcription is the interaction between the RV-G and RV M protein (RV-M). The RV-M is known as an element that regulates the balance of viral transcription and replication [31, 32]. In an RV-infected cell, the RV-M binds to the RV-G and changes its distribution within the cell depending on the presence of the RV-G [33]. It also has been reported that the combination of the RV-M and RV-G affect viral transcription [34]. It is possible that the lack of either the RV-G or the interaction between the RV-G and RV-M might have caused the difference in the transcription activity observed in this study.

We examined the viral transcription and mRFP expression level in this study, but have yet to find any clear correlation between the mRNA expression level and that of protein. In fact, we found that the expression level of mRFP in rHEP5.0-ΔG-mRFP-BPB-infected cells was higher than that of rHEP5.0-ΔG-mRFP- infected cells at 3 and 6 dpi, despite the differences of amounts of mRFP-coding mRNA in those cells not being significant. It is possible that the significant differences of gene-coding mRNA we found in this study do not directly link to the expression level of the protein coded by the mRNA. Quantifying the rabies proteins and examining the correlation between the protein expression and mRNA level at different time points would be important to understand the gene expression mechanism of the rabies virus. This may also help us understand the discrepancy between the level of L-gene-coding mRNA and other viral-gene-coding mRNAs in rHEP5.0-ΔG-mRFP- and rHEP5.0-ΔG-mRFP-BPB-infected cells at 6 dpi.

The ΔG-RV is a powerful tool to investigate the organization and function of neural circuits since it gives investigators the ability to genetically target initial infection to particular neurons and control trans-synaptic propagation [35–37]. This can be achieved by pseudotyping the virus and supplying the G gene within the initially infected neurons [11]. This viral vector is also known to have a high transgene expression level, which enables the labeling of detailed structures, such as spines, and to manipulate neural activity using optogenetic tools [12]. However, the overexpressed transgene can be harmful to infected cells [38, 39]. We previously reported that rHEP5.0-ΔG-mRFP, which shows high transgene expression, infected neurons exhibiting cellular degeneration *in vivo* 21 dpi [13]. It is preferable to develop a ΔG-RV that shows an appropriate transgene expression level to keep the infected cells alive for a long period. The findings we explained in this study enable investigators to design a useful viral vector that shows controlled desirable transgene expression level in applications. We have shown that after G-gene deletion, viral transcription and gene expression are significantly enhanced by increased viral polymerase L-gene expression. This means that investigators can control the viral gene expression via the expression level of the L gene. Finke et al. previously showed that the expression level of the L gene changes depending on the length of the sequence of the intergenic region (IGR) between the G gene and L gene [14]. A shorter IGR in front of the L gene causes higher viral gene expression via higher L-gene expression and a longer IGR causes lower viral gene expression via lower L-gene expression. This enables investigators to design a ΔG-RV vector that shows the restrained gene expression via a longer sequence of IGR in front of the L gene for long expression. On the other hand, investigators also can design a ΔG-RV vector that shows a more rapid and higher gene expression via a shorter sequence of IGR. Such modification may also be useful in designing a G-gene-intact RV vector, as previously described by Finke et al. [14]. The transgene expression level of the G-gene-intact RV vector is relatively low; thus, the signal of the expressed fluorescent protein must be amplified by immunostaining in a tracing study [40, 41]. The development of a G-gene-intact RV vector with a high transgene expression level will enable investigators to omit the immunostaining procedure and further examine the detailed structure of the targeted neurons. We also showed that the presence of the RV-G weakens viral transcription and gene expression. The most likely reason of the weakening is the toxicity of the RV-G, but the precise mechanism is unclear. Further investigation is required to understand the function of the RV-G in viral gene expression.

In this study, we used RV vector variants of the HEP strain which is a highly attenuated RV strain. We previously demonstrated the utility of this RV vector, which has transgenes between N and P gene in their genome, in neural circuits tracing [40, 41]. Recently, the SAD B19 strain based ΔG-RV, which has transgenes between the M and L gene, is mainly used for neural circuits analysis. It is not clear whether our findings in this study are common with the SAD B19 strain based RV vector or a native RV. Future experiments using these RV would be necessary to understand the common mechanism of gene expression and the disparity derived from the differences of the strains or the transgene insertion.

## Supporting Information

S1 Fig Northern blot analysis of viral transcripts in NA cells. Total RNAs were isolated 1, 3, and 6 dpi. The bands of N-, mRFP-, P-, M-, L-, CVS-G-, and BPB-gene-related RNAs were detected using DIG-labeled gene-specific proves and chemiluminescent substrate CSPD. The luminescent images were taken with different exposure times for each lane. RNA constructions were estimated from the size of transcripts, and are shown to the right of the images.
(TIF)

S1 Table Proportional percentages of the band intensities on the Northern blots. The Proportional percentages of the band intensities on the Northern blots were calculated. The percentages of gene-coding mRNA in gene-related RNA (Table 2) were estimated based on these values.
(DOCX)

S2 Table Relative amounts of the viral-gene-coding RNAs. These quantitative data of amounts of the viral-gene-coding RNAs were shown as the graphs in Fig 3 (mean ± SEM). The amounts were collected for “the mean value of the amounts of L-gene-coding mRNA of rHEP5.0-CVSG-mRFP-infected cells 1 dpi” to equal 1.
(DOCX)
