# Supplementary figures and images for: Effects of G-gene Deletion and Replacement on Rabies Virus Vector Gene Expression
Source: PLoS One. 2015 May 29;10(5):e0128020. doi: 10.1371/journal.pone.0128020 (PMC4449044; doi:10.1371/journal.pone.0128020)

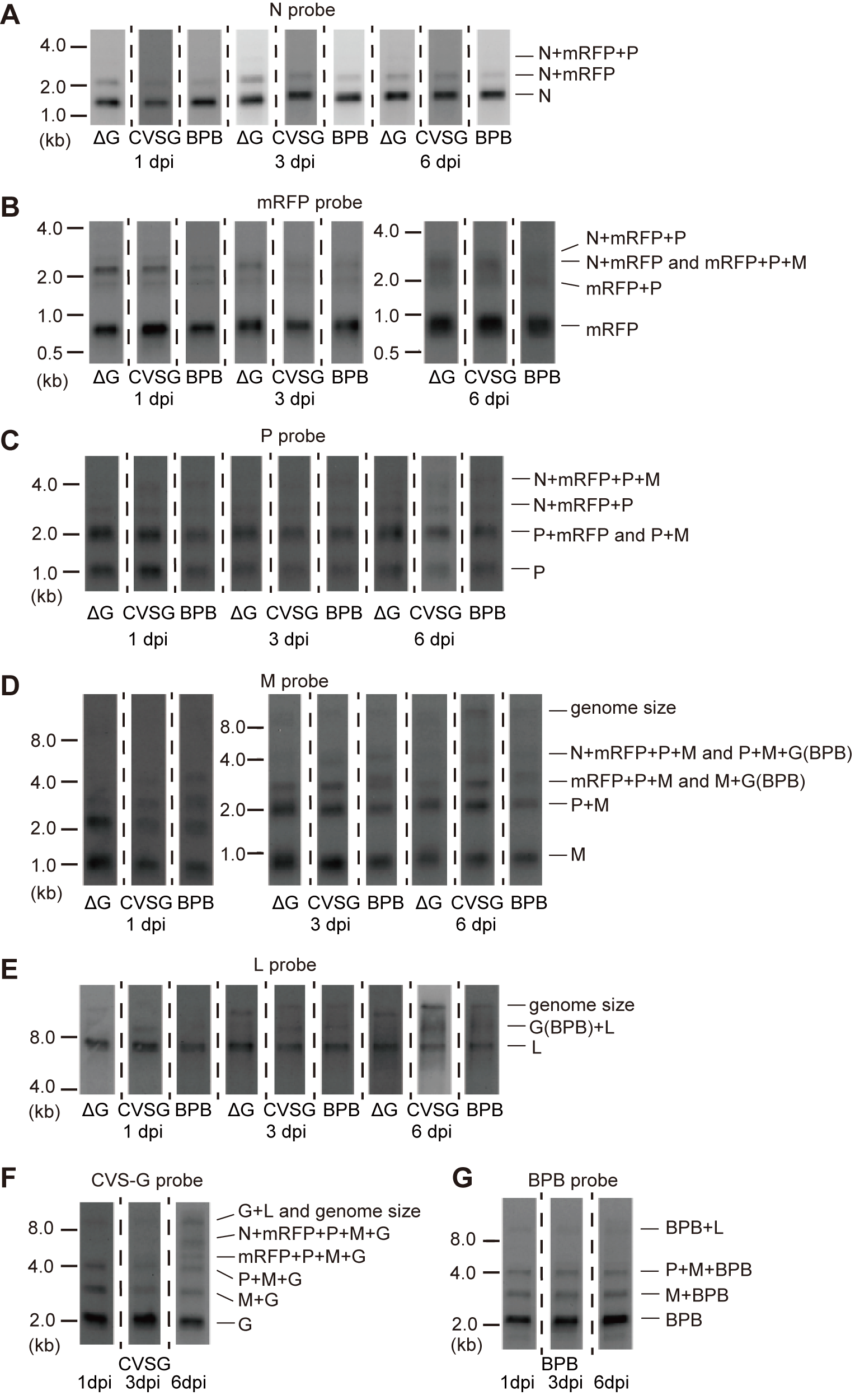

Supplement: S1 Fig — Total RNAs were isolated 1, 3, and 6 dpi. The bands of N-, mRFP-, P-, M-, L-, CVS-G-, and BPB-gene-related RNAs were detected using DIG-labeled gene-specific proves and chemiluminescent substrate CSPD. The luminescent images were taken with different exposure times for each lane. RNA constructions were estimated from the size of transcripts, and are shown to the right of the images. (TIF) [file pone.0128020.s001.tif]
