# Supplementary material for: Effects of G-gene Deletion and Replacement on Rabies Virus Vector Gene Expression
Source: PLoS One. 2015 May 29;10(5):e0128020. doi: 10.1371/journal.pone.0128020 (PMC4449044; doi:10.1371/journal.pone.0128020)
Supplement: S1 Table — The Proportional percentages of the band intensities on the Northern blots were calculated. The percentages of gene-coding mRNA in gene-related RNA (Table 2) were estimated based on these values. (DOCX) [file pone.0128020.s002.docx]

**Table S1**. **Proportional percentages of the band intensities on the Northern blots**

| Probes | Estimated constructions | 1dpi | | | 3dpi | | | 6dpi | | |
| --- | --- | --- | --- | --- | --- | --- | --- | --- | --- | --- |
|  |  | ΔG | CVSG | BPB | ΔG | CVSG | BPB | ΔG | CVSG | BPB |
| N probe | N | 79.4 (%) | 81.8 | 88.2 | 74.9 | 83.6 | 86.2 | 81.9 | 85.1 | 89.1 |
|  | N+mRFP | 18.9 | 16.3 | 10.8 | 23.0 | 15.0 | 12.6 | 16.6 | 14.0 | 10.1 |
|  | N+mRFP+P | 1.7 | 1.8 | 1.1 | 2.0 | - | - | - | - | - |
| mRFP probe | mRFP | 65.0 | 75.0 | 79.5 | 73.1 | 76.6 | 78.4 | 68.4 | 70.3 | 74.7 |
|  | mRFP+P | 8.8 | 7.7 | 8.8 | 10.7 | 10.4 | 11.2 | 10.7 | 10.3 | 13.8 |
|  | N+mRFP and mRFP+P+M | 23.7 | 15.4 | 9.8 | 15.2 | 11.5 | 9.1 | 19.9 | 18.3 | 10.9 |
|  | N+mRFP+P | 2.5 | 1.9 | 1.8 | - | - | - | - | - | - |
| P probe | P | 40.9 | 46.0 | 45.3 | 37.1 | 36.8 | 34.4 | 33.9 | 25.5 | 37.5 |
|  | mRFP+P and P+M | 47.0 | 41.7 | 42.8 | 51.0 | 49.1 | 45.4 | 49.5 | 48.6 | 42.9 |
|  | N+mRFP+P | 8.3 | 6.0 | 4.2 | 8.6 | 7.5 | 9.0 | 11.2 | 12.3 | 8.9 |
|  | N+mRFP+P+M | 3.7 | 6.3 | 7.7 | 3.3 | 6.6 | 11.2 | 5.5 | 13.7 | 10.7 |
| M probe | M | 50.5 | 55.8 | 42.2 | 50.9 | 48.3 | 44.7 | 50.5 | 42.9 | 51.2 |
|  | P+M | 35.6 | 25.6 | 27.1 | 34.2 | 28.8 | 25.5 | 33.4 | 28.9 | 24.9 |
|  | mRFP+P+M and M+G | 10.4 | 13.0 | 20.2 | 10.7 | 15.4 | 20.8 | 9.9 | 15.1 | 15.4 |
|  | N+mRFP+P+M and P+M+G(BPB) | 0.8 | 4.9 | 8.7 | 2.9 | 6.4 | 8.3 | 4.7 | 7.8 | 6.6 |
|  | genome size | 2.6 | 0.7 | 1.7 | 1.3 | 1.0 | 0.8 | 1.4 | 5.3 | 1.8 |
| L probe | L | 96.0 | 83.2 | 86.5 | 86.9 | 69.7 | 72.0 | 87.8 | 58.7 | 73.0 |
|  | G(BPB)+L | 2.3 | 15.1 | 13.5 | 6.4 | 23.3 | 23.9 | 7.4 | 18.0 | 16.6 |
|  | genome size | 1.7 | 1.7 | - | 6.7 | 7.0 | 4.1 | 4.8 | 23.2 | 10.4 |
| G probe | G | - | 57.0 | - | - | 68.8 | - | - | 58.6 | - |
|  | M+G | - | 19.5 | - | - | 12.8 | - | - | 12.7 | - |
|  | P+M+G | - | 13.0 | - | - | 10.9 | - | - | 8.1 | - |
|  | mRFP+P+M+G | - | 3.4 | - | - | 3.1 | - | - | 4.0 | - |
|  | N+mRFP+P+M+G | - | 7.1 | - | - | 4.5 | - | - | 10.1 | - |
|  | G+L and genome size | - | - | - | - | - | - | - | 6.5 | - |
| BPB probe | BPB | - | - | 68.4 | - | - | 65.1 | - | - | 71.4 |
|  | M+BPB | - | - | 18.5 | - | - | 19.9 | - | - | 16.8 |
|  | P+M+BPB | - | - | 10.6 | - | - | 13.1 | - | - | 10.5 |
|  | BPB+L | - | - | 2.4 | - | - | 1.9 | - | - | 1.3 |
