# Supplementary material for: Effects of G-gene Deletion and Replacement on Rabies Virus Vector Gene Expression
Source: PLoS One. 2015 May 29;10(5):e0128020. doi: 10.1371/journal.pone.0128020 (PMC4449044; doi:10.1371/journal.pone.0128020)
Supplement: S2 Table — These quantitative data of amounts of the viral-gene-coding RNAs were shown as the graphs in Fig 3 (mean ± SEM). The amounts were collected for “the mean value of the amounts of L-gene-coding mRNA of rHEP5.0-CVSG-mRFP-infected cells 1 dpi” to equal 1. (DOCX) [file pone.0128020.s003.docx]

**Table S2**. **Relative amounts of the viral-gene-coding RNAs**

| Gene | Date | rHEP5.0-ΔG-mRFP | rHEP5.0-CVSG-mRFP | rHEP5.0-ΔG-mRFP-BPB |
| --- | --- | --- | --- | --- |
| N | 1dpi | 26.9 ± 3.6 | 10.4 ± 1.0 | 17.7 ± 1.2 |
|  | 3dpi | 119.5 ± 12.5 | 53.7 ± 7.6 | 91.3 ± 12.9 |
|  | 6dpi | 170.5 ± 18.5 | 52.2 ± 5.4 | 95.5 ± 11.3 |
| mRFP | 1dpi | 9.9 ± 1.9 | 5.5 ± 0.6 | 7.7 ± 0.9 |
|  | 3dpi | 77.0 ± 8.5 | 41.2 ± 6.3 | 61.0 ± 8.5 |
|  | 6dpi | 141.8 ± 16.6 | 54.1 ± 8.3 | 73.6 ± 4.8 |
| P | 1dpi | 11.7 ± 1.7 | 4.6 ± 0.4 | 7.1 ± 0.4 |
|  | 3dpi | 39.5 ± 3.0 | 23.7 ± 3.0 | 26.5 ± 3.2 |
|  | 6dpi | 70.0 ± 4.9 | 21.6 ± 2.9 | 32.7 ± 2.2 |
| M | 1dpi | 3.2 ± 0.4 | 2.1 ± 0.1 | 2.1 ± 0.1 |
|  | 3dpi | 11.0 ±1.0 | 7.8 ± 1.2 | 9.2 ± 1.3 |
|  | 6dpi | 30.4 ± 3.1 | 10.6 ± 1.8 | 18.8 ± 1.3 |
| G(BPB) | 1dpi | - | 1.9 ± 0.2 | 2.5 ± 0.4 |
|  | 3dpi | - | 13.1 ± 2.2 | 9.1 ± 1.5 |
|  | 6dpi | - | 6.0 ± 1.3 | 20.1 ±5.5 |
| L | 1dpi | 3.8 ± 0.4 | 1.0 ± 0.1 | 1.4 ± 0.2 |
|  | 3dpi | 18.2 ± 3.9 | 7.4 ± 1.5 | 7.2 ± 1.2 |
|  | 6dpi | 10.5 ± 1.7 | 8.2 ± 1.9 | 4.2 ± 0.6 |
| Total | 1dpi | 55.5 ± 7.5 | 25.5 ± 2.0 | 38.5 ± 2.0 |
|  | 3dpi | 265.2 ± 26.1 | 146.8 ± 21.2 | 204.4 ± 26.8 |
|  | 6dpi | 423.2 ± 34.5 | 152.7 ± 19.9 | 244.9 ± 12.2 |
